# Rapid and sensitive biomarker detection using molecular imprinting polymer hydrogel and surface-enhanced Raman scattering

**DOI:** 10.1098/rsos.171488

**Published:** 2018-01-24

**Authors:** Shaona Chen, Lijing Dong, Min Yan, Zhongxu Dai, Chenghua Sun, Xin Li

**Affiliations:** 1 College of Materials and Chemical Engineering, China Three Gorges University, Yichang, Hubei 443002, People's Republic of China; 2 Hubei Key Laboratory of Natural Products Research and Development, China Three Gorges University, Yichang, Hubei 443002, People's Republic of China; 3 Department of Chemistry and Biotechnology, Faculty of Science, Engineering and Technology, Swinburne University of Technology, Hawthorn, Victoria 3122, Australia; 4 School of Chemistry and Chemical Engineering, Harbin Institute of Technology, Harbin 150001, People's Republic of China

**Keywords:** biomarker detection, molecular imprinting polymer hydrogel, surface-enhanced Raman scattering

## Abstract

Biomarkers are important biochemical indicators, which could be used for identification, early diagnosis and monitoring of diseases during the course of treatment. However, biomarker diagnosis has some shortcomings such as requiring a large amount of samples, long test time and high cost, which seriously influences the correctness and timely treatment to patients. Here, a relatively fast and efficient plasmonic hot spot-localized surface imprinting of Ag spheres using biomarker template immobilization and hydrogel copolymerization is described. The technique takes a fine control of the imprinting process at the nanometre scale and provides a biosensor with high sensitivity. Proof of the opinion is established by detection of biomarker using surface-enhanced Raman scattering (SERS) spectroscopy. This work represents a valuable step towards SERS with biomarkers for cost-saving and time-saving diagnostic assay. It is expected that the new surface imprinted hydrogel plasmonic material can drive possibilities in advancing application of biomarkers in plasmonic biosensors.

## 1. Introduction

In recent years, biomarkers have been widely used in clinical diagnosis for the most crucial and important diseases. Many analytical testing methods, such as enzyme-linked immunosorbent assay, are very selective and accurate, but still have several disadvantages such as destructiveness, expensiveness and time consuming. The challenge is thus to find a sensitive, accurate, simple, rapid and low-cost method for detecting biomarkers in medical services.

Surface-enhanced Raman scattering (SERS), as a powerful analytical tool, has attracted considerable attention for possessing fingerprint information, high sensitiveness and requiring small amounts of samples for testing [1–3]. It is a non-destructive and non-invasive technique that can produce strongly enhanced Raman signals of adsorbed molecules on noble metal surfaces, such as Ag, Au and Cu [4]. Among them, the SERS enhanced effect of Ag is the best [5]. However, bare noble metals are easily oxidized, such that their SERS stability will be degraded [6,7].

Molecularly imprinted polymers (MIPs), as artificial receptors, exhibit high selectivity and absorbability for target template because of specifically recognizing cavities in the polymers. MIPs also have many advantages, such as low cost, reusability, stability, adsorption and selectivity. To further improve stability, sensitivity and selectivity of Raman signals, MIPs have been introduced to integrate on the surface of a metal substrate. Xue *et al*. have fabricated a bisphenol A MIP layer on the surface of Au nanoparticles for highly selective detecting of target molecules [8]. Bompart *et al*. [9] have prepared a chemical nanosensor for detecting (*S*)-propranolol molecules based on MIP-SERS. Holthoff *et al*. [10] have reported that imprinted nanosensors were prepared for detecting 2,4,6-trinitrotolunene molecules by SERS. Very recently, we also demonstrated that hybrid substrates have high sensitivity of Raman signals for target molecules based on SERS–MIP [11–13]. Researchers also found the excellent properties of the substrate being used to improve the Raman signals combining SERS with MIP [14]. Nonetheless, this new technology is seldom applied in biomedical diagnosis. Biosensing platforms based on SERS and MIP hold enormous potential to provide sensitive, undamaged, rapid and low-cost diagnostic tools.

To apply the new technology of SERS and MIP in the medical diagnosis field, we adopt the MIP hydrogel (MIPH) method. Hydrogel, which has excellent biocompatibility, is introduced into the MIP method to synthesize non-toxic polymers in aqueous solution for preserving bioactivity of the biomarker.

**Figure 1.**
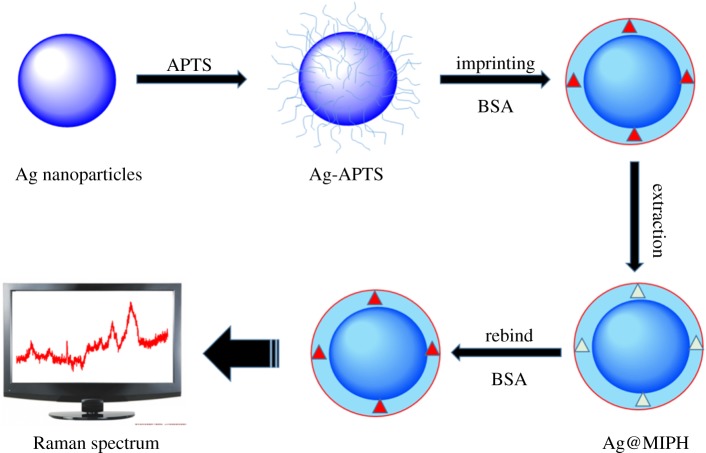
Schematic illustration of the prepared processing of Ag@MIPH for detecting BSA by SERS.

In this work, bovine serum albumin (BSA) is employed as target analytes. Our approach uses Ag spheres with an MIPH layer to super-sensitively detect BSA molecules. The preparation process of Ag@MIPH is shown in figure 1. Specific steps are as follows: first, Ag spheres were obtained by the chemical deposition method at room temperature and then modified by 3-aminopropyltriethoxysilane (APTS) to synthesize Ag-APTS nanoparticles. Second, BSA as the target template was polymerized on the surface of Ag-APTS nanoparticles to synthesize the MIPH layer, and then the BSA molecules were removed by 5% sodium dodecyl sulfate (SDS) solution to leave memory cavities. Through many experiments, we find that the prepared Ag@MIPH could rapidly and sensitively detect the Raman signals of BSA. The results show that the Ag@MIPH nanoparticles can improve the sensitivity of BSA Raman signals to 10^−8^ M. In brief, we have fabricated the core-shell Ag@MIPH substrate to detect Raman signals of BSA molecules, and the prepared biosensing platform Ag@MIPH nanoparticles have shown excellent performance.

## 2. Material and methods

### 2.1. Materials

Silver nitrate (AgNO_3_), ammonium persulfate (APS) and ethanol were purchased from the Tianda Chemical Reagent (Shanghai, China). SDS was purchased from Guoyao Chemical Reagent (Shanghai, China). Acrylamide (AM) was obtained from Guangfu Chemical Industry (Tianjin, China). APTS and *N*,*N*′-methylenebisacrylamide were obtained from Alfa Aesar. BSA, bovine haemoglobin (BHb) and fibrinogen were purchased from Sigma-Aldrich (St Louis, MO, USA). *N*,*N*,*N*′,*N*′-tetramethylethylenediamine (TEMED) was obtained from Alfa Aesar. Ascorbic acid (VC) and polyvinyl pyrrolidone (PVP) were purchased from the Tianjin Damao Chemical Reagent Factory (Tianjin, China).

### 2.2. Synthesis of Ag nanoparticles

Ag nanoparticles were synthesized by using the chemical deposition method at room temperature. First, AgNO_3_ (0.425 g) and PVP (0.85 g) were dissolved into 244 ml ultrapure water solution. Then, 4 ml of VC ultrapure water solution (containing 0.7 g of VC) was quickly injected into the above mixed solution, and the mixture was reacted for 15 min under stirring. At this stage, the colour of the mixture solution changed from dark to claybank. Ag nanoparticles were washed several times with ethanol and water by centrifugation. The product was dried under a vacuum at 40°C for 24 h.

### 2.3. Synthesis of Ag-APTS nanoparticles

Ag nanoparticles (200 mg) were sonicated for 20 min in 400 ml of ethanol–ultrapure water (2 : 1, v/v). The mixture was stirred at 40°C for 24 h with the addition of 2 ml of APTS under nitrogen protection. The obtained product was dried under a vacuum at 40°C for 24 h.

### 2.4. Synthesis of Ag@MIPH nanoparticles

A total of 80 mg of BSA (template molecules), 285 mg of AM (functional monomer), 15 mg of *N*′,*N*′-methylenebisacrylamide (functional monomer) and 100 µl of TEMED (cross-linking agent) were dissolved in 10 ml of phosphate buffer solution. After 80 mg of Ag-APTS nanoparticles were dispersed in the above solution under nitrogen, 1 ml of APS solution was dropped into the mixture under stirring. The mixed solution was magnetically stirred for 40 min at room temperature. The grey white product was washed with 5% SDS solution until no BSA molecules were detected in the washing solution. Finally, core-shell Ag@MIPH nanoparticles were dried under vacuum at 40°C for 24 h.

### 2.5. Characterization

The shape and structure of the particles were examined with a transmission electron microscope (TEM; Tecnai G20 Philip) and scanning electron microscopy (SEM; FEI HELIOS NanoLab 600i). Phase structure of products was characterized by X-ray diffractometer (XRD; Shimadzu XRD-6000) with Cu K*α* radiation (*λ* = 1.5406 Å). The structural groups of the products were analysed by Fourier transform infrared spectrometer (FT-IR, Avatar 360). A laser at a wavelength of 633 nm and the Raman spectra were collected with a Renishaw in a Via micro-Raman spectroscopy system. The laser power and accumulation were 0.1 mW and 1 s.

## 3. Results

**Figure 2.**
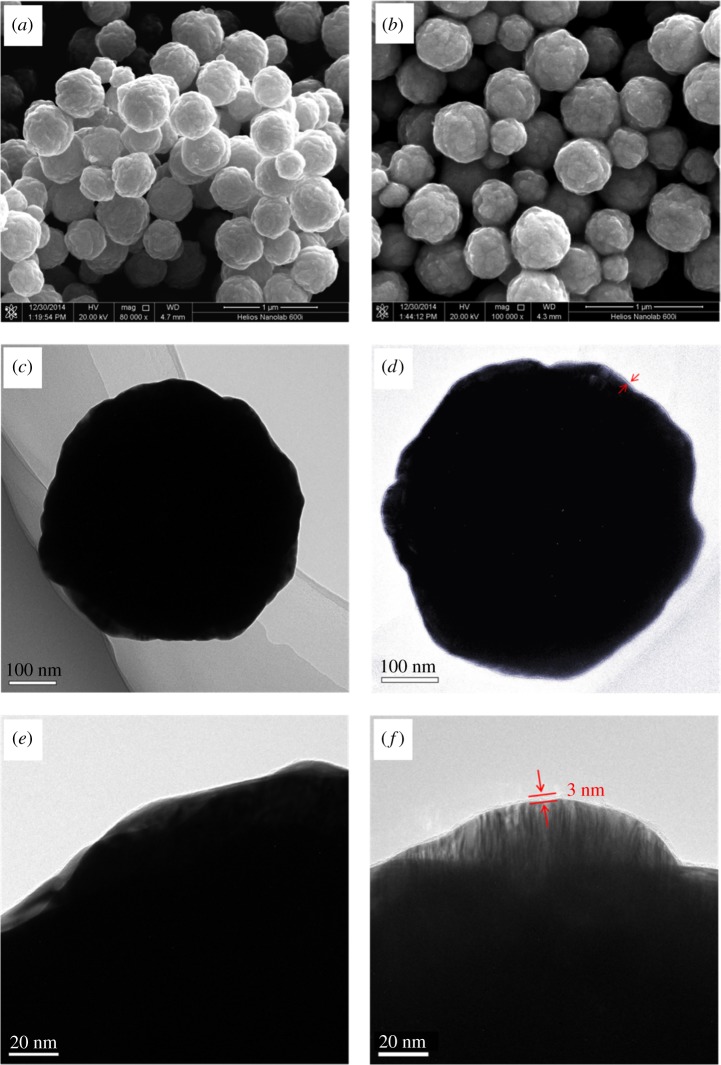
SEM images of Ag nanoparticles (*a*) and Ag@MIPH (*b*). Low- and high-magnification TEM images of Ag nanoparticles (*c*,*e*) and Ag@MIPH (*d*,*f*).

In this study, the morphology and structure of the Ag spheres and Ag@MIPH were characterized by SEM and TEM (figure 2). A chemical deposition method was employed to synthesize Ag nanoparticles, which can be clearly observed in figure 2*a*,*c*. The SEM and TEM images show that the Ag nanoparticles are spheric morphology, while the shape is regular and the particle size is homogeneous. Figure 2*e* shows high-magnification TEM image of Ag nanoparticles, and the surface of Ag nanoparticle is clean. The SEM and TEM images of Ag@MIPH nanoparticles reflect the nanosheet structure in figure 2*b*,*d*, and the MIPH shell is about 3 nm which could enhance the stability of the Ag@MIPH substrate coated on the surface of Ag core in figure 2*f*. The ultrathin MIPH layer could improve the sensitivity of BSA Raman signals and prevent the oxidation of the Ag spheres.

**Figure 3.**
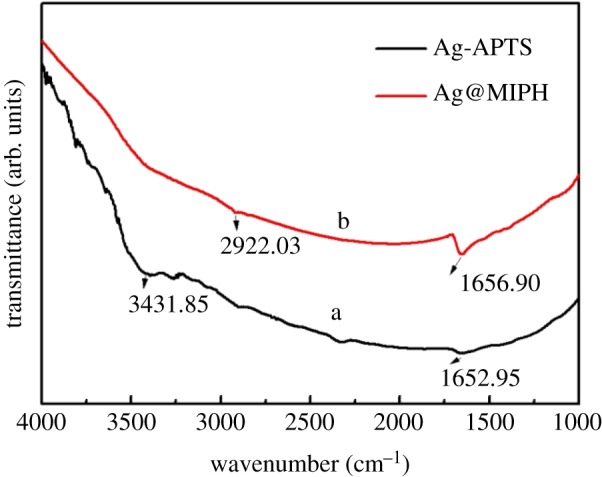
FI-IR spectra of Ag-APTS (a) and Ag@MIPH (b).

The FI-IR spectra of Ag-APTS and Ag@MIPH are presented in figure 3. The vibration peaks at approximately 1652.95 and 3431.85 cm^−1^ are attributed to the –N-H vibration and stretch-bending modes from Ag-APTS, respectively (figure 3, line a). The peak at 2922.03 cm^−1^ is associated with the stretch of carboxylic acid groups, and the peak at 1656.90 cm^−1^ corresponds to –N-H of BSA on the Ag@MIPH (figure 3, line b). It further indicates that Ag@MIPH has been successfully prepared.

**Figure 4.**
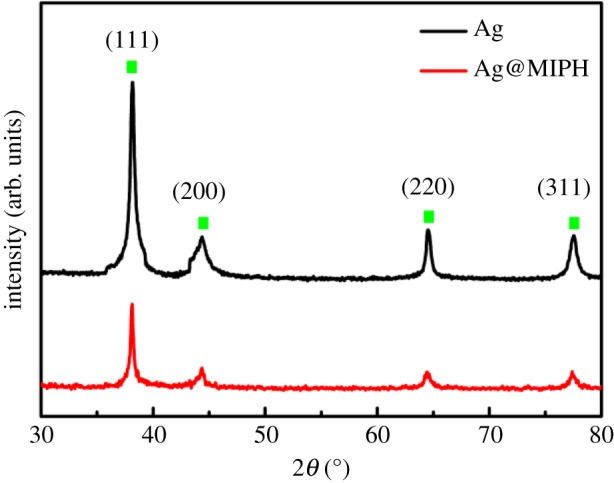
XRD patterns of Ag and Ag@MIP nanoparticles.

The XRD patterns of Ag spheres and Ag@MIPH are presented in figure 4. As displayed, Ag spheres and Ag@MIPH show four strong diffraction peaks at 2*θ* = 38.14°, 44.24°, 64.55° and 77.42°, which correspond to (111), (200), (220) and (311) crystal planes of cubic Ag in the card (JCPDS file no. 04-0783). After MIPH shell has been coated on the surface of Ag spheres, the intensity of Ag@MIPH diffraction peaks became weaker. This reveals that polymerization of Ag spheres does not lead to their phase changes, suggesting successful synthesis of Ag@MIPH.

**Figure 5.**
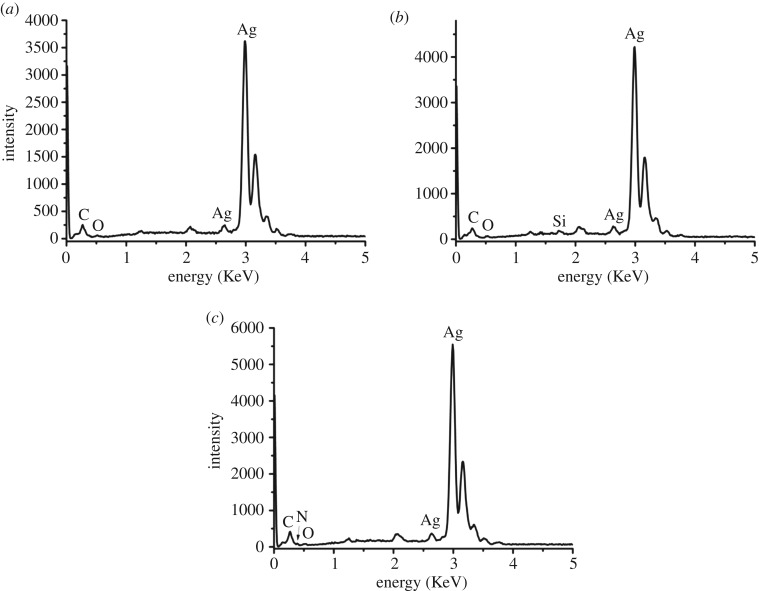
EDS spectra of (*a*) Ag nanoparticles, (*b*) Ag-APTS and (*c*) Ag@MIPH.

EDS spectra of Ag nanoparticles, Ag-APTS and Ag@MIPH are presented in figure 5. As displayed in figure 5*a*, the Ag element appears in the EDS spectrum of Ag nanoparticles, suggesting the formation of Ag spheres. The EDS spectrum of Ag-APTS shows the signals of Si, Ag elements in figure 5*b*, and this indicates that the surface of Ag sphere has been modified by APTS. After MIPH layer was coated on the surface of Ag-APTS nanoparticles, N and Ag elements are demonstrated in the EDS spectrum of Ag@MIPH in figure 5*c*. N and Ag elements are derived from the BSA in MIPH layer and Ag spheres, respectively. The results suggest the successful preparation of Ag@MIPH.

BSA is known to be rich in α-helix structure, and it has no protein-bound chromophore group, the SERS-active groups are composed of aromatic side chains and amide groups such as tryptophan (Trp), tyrosine (Try) and phenylalanine (Phe). As one of the simple biomarkers, BSA, was chosen as a sensitive and selective probe to reveal the SERS activity of Ag@MIPH and Ag nanoparticles.

**Figure 6.**
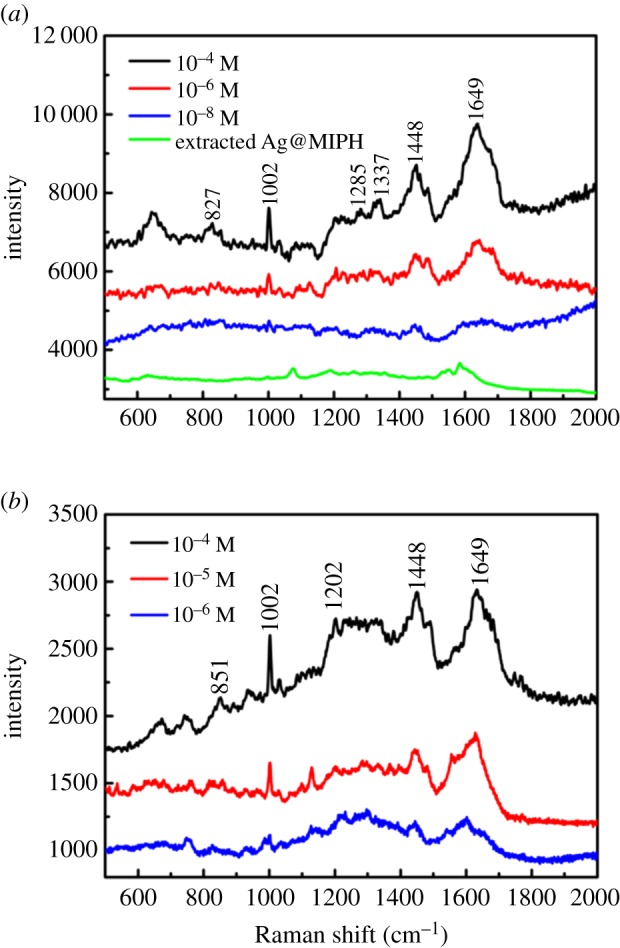
SERS spectra of BSA in different concentrations on (*a*) Ag@MIPH and (*b*) Ag nanoparticles.

In our experiment, Ag@MIPH and Ag nanoparticles were, respectively, soaked in the different concentrations of BSA solution for 2 h, and then the mixed liquid was dropped on the centre of a piece of glass to dry at room temperature. The SERS spectra of BSA collected from the dry sample are depicted in figure 6. Since the BSA molecules near the surface of the substrate would be enhanced, the peaks of adsorbed biomarker SERS spectra may be moved, and the resulting Raman spectra of BSA almost match the previous report [15]. The SERS spectra of Ag@MIPH with different concentrations of BSA solution are shown in figure 6*a*. The strong band at approximately 1649 cm^−1^ is assigned to amide I vibration. The characteristic band at 1448 cm^−1^ is recognized as a characteristic C–H vibration peak. The bands at approximately 1337 and 1285 cm^−1^ are associated with Trp and amide III groups. The Raman bands for BSA are assigned to the Phe and Tyr vibrations located at 1002 and 827 cm^−1^, respectively. As expected, the SERS intensity increased with the increase in BSA concentration. Obviously, the sharp peak at 1002 cm^−1^, as a quantitative measurement of BSA, appears in BSA solutions of different concentrations and can be clearly observed even at a concentration of 10^−8^ M. According to the previous report [11], the enhancement factor was estimated to be 5.56 × 10^5^ mol l^−1^ using a BSA peak at 1002 cm^−1^. In figure 6*b*, the SERS spectra of Ag with different concentrations of BSA have been shown. Compared with the SERS spectra of Ag@MIPH, the characteristic bands at 1285 and 1337 cm^−1^ have become relatively weak, while the band at 1202 cm^−1^ has become more obvious. The minimum concentration of BSA solution detected with the Ag spheres is 10^−6^ M. As can be seen from figure 6, the limiting detectable concentration of Ag@MIPH has been improved by two orders of magnitude when compared with the Ag nanoparticles. The results show that the MIPH layer coated on the surface of Ag nanoparticles, as an active SERS substrate, can improve the SERS activity and has selective capacity for detecting the probe molecules.

**Figure 7.**
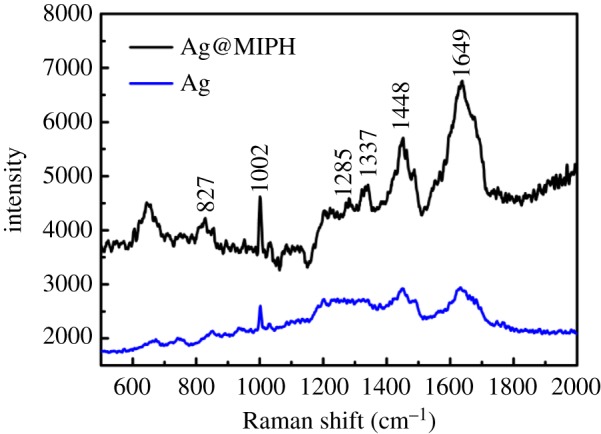
SERS spectra of Ag@MIPH and Ag nanoparticles in 10^−4^ M BSA solution.

**Figure 8.**
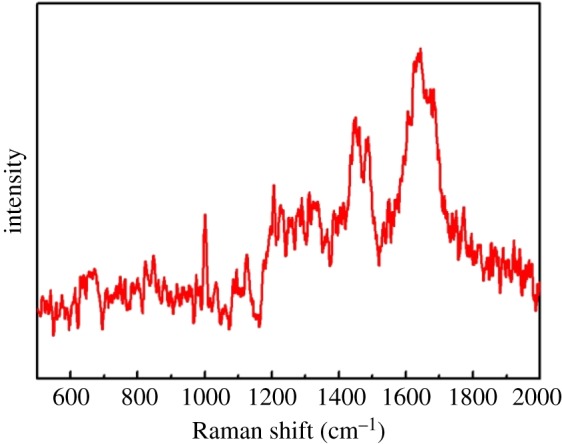
SERS spectra after competitive binding experiments: 10^−4^ M mixed solution of BSA, BHb and fibrinogen.

We compare the SERS spectra of Ag@MIPH and Ag nanoparticles in 10^−4^ M BSA solution as shown in figure 7. As expected, the Raman signals from the Ag@MIPH are stronger than the signals from the pure Ag nanoparticles. This is because the imprinting cavity of MIPH can selectively absorb BSA to improve its concentration on the surface of Ag@MIPH. To evaluate the selectivity of Ag@MIPH towards BSA, BHb and fibrinogen were selected as interfering substances owing to their enormous intensity enhancement. As shown in figure 8, no characteristic Raman peaks could be observed for interferents.

## 4. Discussion

The good SERS performance of Ag@MIPH can be attributed to both the MIPH and core-shell structure. The MIPH on the surface of Ag@MIPH has major synergistic effects. First, the MIPH has a swelling effect in the solvent, such that BSA could enter into the three-dimensional imprinted cavity of MIPH and finally reach the surface of Ag-core owing to ‘gate effect’. BSA can get into the MIPH imprinted cavity to improve the concentration on the surface of the Ag@MIPH in solvent. Second, the core-shell structure possesses a long-range effect of localized surface plasmon resonance from the electromagnetic mechanism (EM). In other words, the SERS signals can be detected further away from the Ag-core. For our Ag@MIPH, an ultrathin MIPH shell which conforms to long-range effect was encapsulated on the surface of the Ag-core; as a result, the signals could be enhanced by high-intensity plasmon-resonant local field. According to the above discussion, the significant SERS enhancement is attributed to the combination of EM effects.

## 5. Conclusion

By combining the technique of molecular imprinting and SERS, we have developed an effective analytical technique for biomarker detection by using a green synthesis method of core-shell Ag@MIPH. The Ag@MIPH, as an excellent SERS-active substrate for detecting biomarkers, plays an important role in this study. Using this technique, we detected the lowest concentration of BSA up to 10^−8^ mol l^−1^. The sensitivity of Ag@MIPH is far superior to that of Ag. From the results, it seems that the Ag@MIPH are very good materials not only for biomarker imprinting, but also for SERS detection of biomarkers. This environmentally friendly approach may take us a step closer to real-world application of the Ag@MIPH hybrids for detecting biomarkers.

## Data accessibility

This article has no additional data.
